# Assessing the Effect of a Pandemic on Emergency Medical Service Response Times and Interventions for Out-of-Hospital Cardiac Arrest

**DOI:** 10.7759/cureus.75736

**Published:** 2024-12-15

**Authors:** Atsuo Nakamura, Masaharu Odo

**Affiliations:** 1 Department of Emergency and Critical Care Medicine, Iizuka City Hospital, Iizuka, JPN

**Keywords:** covid-19, emergency medical services, intubation, out-of-hospital cardiac arrest, pandemics, patient care

## Abstract

Objectives: The coronavirus disease 2019 (COVID-19) pandemic has significantly disrupted emergency medical service (EMS) prehospital care for patients with out-of-hospital cardiac arrest (OHCA), necessitating a thorough assessment of its effects on prehospital time and emergency interventions. Therefore, we aimed to analyze the changes in EMS operations before and after the onset of the pandemic and their potential effects on patient care.

Methods: We retrospectively reviewed OHCA cases between January 2017 and December 2022, categorizing them into pre-pandemic and pandemic phases. We examined the prehospital time from call intake to hospital arrival, analyzing time segments in detail (on-scene arrival, patient contact, loading, and departure) and procedural frequency/location. Changes in prehospital time, requests for hospital admission, laryngeal tube insertion, and venous line establishment were assessed using a multivariate analysis.

Results: Among the 925 OHCA cases, the pandemic phase (n = 467) experienced a 3-minute average prehospital delay compared with the pre-pandemic phase (n = 458) (P < 0.0001). Specifically, on-scene arrival time (adjusted odds ratio (aOR): 2.06; 95% confidence interval (CI): 1.36-3.11), laryngeal tube insertions (aOR: 3.2; 95% CI: 2.1-4.9), and post-transport venous access placements (aOR: 1.67; 95% CI: 1.06-2.63) increased. Hospital admission requests also increased significantly (aOR: 9.5; 95% CI: 2.78-32.7).

Conclusion: These findings indicate that pandemic conditions delayed EMS responses and altered clinical practices, highlighting the urgent need for EMS system enhancements to improve on-site interventions. Therefore, addressing these challenges, particularly through strategies that expedite early adrenaline administration, is essential for optimizing patient outcomes.

## Introduction

The coronavirus disease 2019 (COVID-19) pandemic, which began in December 2019, has significantly affected healthcare systems globally, with a reported decline in the survival rates of patients with out-of-hospital cardiac arrest (OHCA). The pandemic-related impact on prehospital emergency care has become a primary concern [1-3]. Notably, at the onset of the pandemic, cardiopulmonary resuscitation (CPR) protocols in Japan require modifications [4-6], raising concerns regarding the potential decline in the quality of emergency medical services (EMSs) for OHCA cases. Previous studies have indicated that the time from emergency call acknowledgment by the fire command center to on-scene arrival of the EMS team is prolonged [1,7-9]; however, to date, no studies have examined in detail the overall time to hospital arrival or changes in EMS interventions. Therefore, in this retrospective, exploratory, observational study, we aimed to evaluate the changes in prehospital time and EMS interventions for OHCA before and during the COVID-19 pandemic.

## Materials and methods

The prehospital EMS system in the study area

As of May 2024, individuals aged ≥ 65 years constituted 29.2% of Japan's total population [10]. The study area was a regional city in Fukuoka Prefecture with a population of 175,000 and an older population exceeding 30%. The Iizuka District Fire Department oversees prehospital care, including emergency transportation, with the Emergency and Critical Care Center and our hospital serving as critical facilities for OHCA patient management. Typical procedures paramedics can perform for OHCA include electrical defibrillation, airway management using tracheal or supraglottic devices, secure venous access, and administration of intravenous adrenaline. However, except for defibrillation, these procedures require specific instructions from a medical control doctor over the phone [11]. In contrast, many other countries allow EMS personnel to administer emergency care under general directives. For OHCA in Japan, guidelines recommend administering adrenaline at the scene as soon as patient contact occurs [12].

Changes in response to OHCA since the onset of the COVID-19 pandemic

In Japan, crucial changes in EMS response to OHCA after the COVID-19 pandemic include the mandatory use of personal protective equipment, conduction of a COVID-19-related medical interview, and prioritization of airway management through tracheal intubation or supraglottic devices. In cases where EMS personnel resuscitate patients with COVID-19 during cardiac arrest, they prioritize securing the airway as soon as possible after contact to minimize the risk of infection, including aerosols.

Ethical considerations

This study was approved by our hospital’s Ethics Committee (study number: 2022-04). The Iizuka City Fire Department provided the Utstein data for this analysis, and written consent was obtained. Data were anonymized by deleting personal identifiers and were not linked to specific individuals.

Study participants

**Figure 1:**
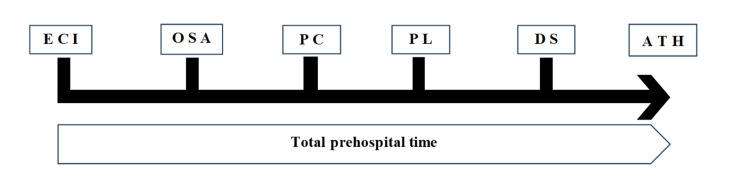
Prehospital time The figure shows the details of prehospital time, and the unit of time is shown in minutes. ECI refers to the point in time at “the acknowledgment of the emergency call by the fire command center.” Abbreviations: ECI, emergency call intake; OSA, on-scene arrival; PC, patient contact; PL, patient loading into ambulance; DS, departure from the scene; ATH, arrival at the hospital This is the original image created by the authors. No further permission is required for its use.

The study included patients with OHCA who received responses from the Iizuka District Fire Department between January 1, 2017, and December 31, 2022. The exclusion criteria were non-transport cases owing to social death, inter-facility transfers, exogenous causes, cases confirmed to be non-resuscitable, and cases where the return of spontaneous circulation (ROSC) was achieved at EMS contact, thereby preventing cardiac arrest during subsequent activities. The EMS procedures analyzed included supraglottic device insertion (laryngeal tubes), establishing intravenous access, and administering adrenaline. The total prehospital time was calculated using the emergency call intake (ECI), on-scene arrival (OSA), patient contact, patient loading into an ambulance (PL), departure from the scene (DS), and arrival at the hospital (ATH), with ECI to ATH defined as the total prehospital time (Figure 1). The period was divided into two phases: three years before and after the pandemic. The pre-pandemic phase is from January 1, 2017, to December 31, 2019, and the pandemic phase is from January 1, 2020, to December 31, 2022. The calculated prehospital times and EMS procedures were compared between the phases. Additionally, the differences in OHCA characteristics, EMS activity details, and outcomes were analyzed using a multivariate analysis.

Statistical analysis

The parameters were recorded as median values (range) or counts (percentages). Wilcoxon rank-sum tests and chi-square tests were used for phase comparisons. Multivariate logistic regression was applied to evaluate the differences in OHCA characteristics and outcomes, adjusting for potential confounders selected for statistical significance and clinical relevance. These confounders included pulseless electrical activity, bystander CPR, witnessed arrest, prehospital time from ECI to OSA and PL to DS, multiple EMS hospital admission requests, laryngeal tube insertion, intravenous access during hospital transport, and prehospital ROSC. Multicollinearity was checked before the analysis. However, the effects of confounders other than the factors used as explanatory variables in this analysis cannot be ruled out. Model adequacy was verified using the Hosmer-Lemeshow test (P = 0.80). Statistical significance was set at two-sided P-values < 0.05. Notably, no missing data were present in the dataset used in this analysis, and no statistical sample size calculations were conducted. The post-hoc powers for detecting the difference in bystander CPR, prehospital time from ECI to OSA, multiple EMS hospital admission requests, laryngeal tube insertion, and intravenous access during hospital transport between the pre-pandemic and pandemic phases are 52.3%, 98.6%, 96.9%, and 96.9%, respectively.

Notably, all statistical analyses were performed using EZR (Saitama Medical Center, Jichi Medical University, Saitama, Japan), a graphical user interface for R (The R Foundation for Statistical Computing, Vienna, Austria). It is a modified version of the R commander (version 2.9-1), designed to add statistical functions frequently used in biostatistics [13].

Patient and public involvement statement 

No patients or members of the public were involved in this retrospective study, which used anonymized data from OHCA cases. The findings will be shared with relevant stakeholders, and future studies will consider patient and public involvement to enhance relevance.

## Results

**Figure 2:**
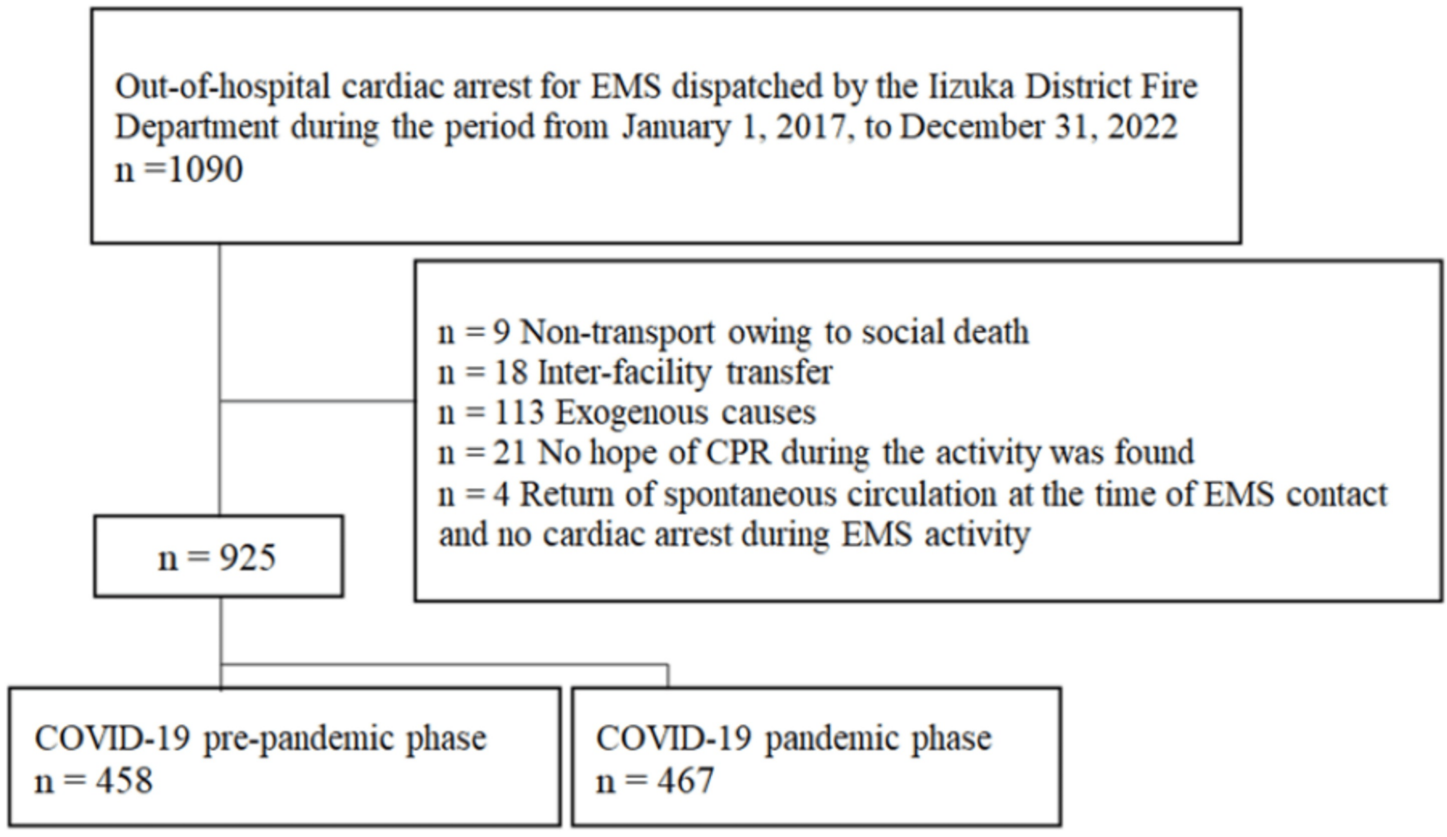
Flowchart depicting the enrollment of patients in the study Abbreviations: EMS, emergency medical services; CPR, cardiopulmonary resuscitation; COVID-19, coronavirus disease 2019

During the study period, 1,090 OHCA cases were observed, of which 925 met the inclusion criteria (Figure 2). Table 1 presents the patient characteristics and prehospital times. The total prehospital time was significantly longer by two minutes during the pandemic phase than during the pre-pandemic phase (P < 0.0001), with ECI to OSA and PL to DS extended by one minute (P < 0.0001) and one minute (P < 0.0001), respectively. Hospital admission requests increased significantly during the pandemic phase (P < 0.0001).

**Table 1:** Patient characteristic and total prehospital time Results are presented as median (range) or actual values (percentages). Abbreviations: ECI, emergency call intake; OSA, on-scene arrival; PC, patient contact; PL, patient loading into ambulance; DS, departure from the scene; ATH, arrival at the hospital; CPR, cardiopulmonary resuscitation; ECG, electrocardiogram; COVID-19, coronavirus disease 2019

	Total	COVID-19	COVID-19	P-value	Statistical value
		Pre-pandemic phase	Pandemic phase		
		2017.01.01 to 2019.12.31	2020.01.01 to 2022.12.31		
Out-of-hospital cardiac arrest	n = 925	n = 458	n = 467		
Age, years	83 (65-106)	82 (8.7)	83 (8.8)	0.25	W= 102294
Sex, male	468 (51%)	231 (50)	237 (51)	0.98	χ^2^= 0.00086226
Witnessed, yes	309 (33%)	141 (31)	168 (36)	0.1	χ^2^= 2.5695
Bystander CPR, yes	409 (44%)	189 (41)	220 (47)	0.08	χ^2^= 2.9679
Initial ECG waveform in cardiac arrest					
Ventricular fibrillation or pulseless ventricular tachycardia	54 (5.8%)	29 (6%)	25 (5%)	0.57	χ^2^= 0.24445
Pulseless electrical activity	257 (28%)	114 (25%)	143 (31%)	0.06	χ^2^= 4.5014
Asystole	614 (66.2%)	315 (69%)	299 (64%)	0.14	χ^2^= 2.1312
Prehospital time					
ECI to ATH (total prehospital time), min	32 (11-100)	31 (11-72)	33 (12-100)	< 0.0001	W= 89934
ECI to OSA, min	9 (3-25)	9 (3-25)	10 (3-22)	< 0.0001	W= 88895
OSA to PC, min	1 (0-9)	1 (1-9)	1 (0-7)	0.83	W= 106498
PC to PL, min	7 (0-43)	6 (1-23)	7 (0-43)	0.38	W= 103352
PL to DS, min	4 (1-52)	3 (1-52)	4 (1-49)	< 0.0001	W= 87123
DS to ATH, min	9 (1-40)	9 (1-36)	9 (1-40)	0.6	W= 105024
Total number of inquiries about hospital admission requests	1 (1-13)	1(1-8)	1 (1-13)	< 0.0001	W= 95753

The EMS interventions and outcome data indicated significant increases in laryngeal tube insertion (P < 0.0001) and adrenaline administration (P = 0.01) during the pandemic phase (Table 2). Orotracheal intubation was not performed for any patient. The time from patient contact to laryngeal tube insertion (P < 0.0001), intravenous access (P < 0.0001), and adrenaline administration (P < 0.0001) was significantly longer during the pandemic phase than during the pre-pandemic phase. The proportion of cases in which these procedures were performed at the scene decreased to 15% (P = 0.0001) and 20% (P = 0.0005) for intravenous access and adrenaline administration, respectively. However, the proportion of cases in which these procedures were conducted during transport increased to 13% (P = 0.01) and 17% (P = 0.01), respectively. Notably, no significant differences in prehospital ROSC or cerebral performance category outcomes were observed.

**Table 2:** Emergency medical service interventions and outcomes Results are presented as median (range) or actual values (percentages). Abbreviations: EMS, emergency medical services; CPC, cerebral performance category; COVID-19, coronavirus disease 2019

	Total	COVID-19	COVID-19	P-value	Statistical value
		Pre-pandemic phase	Pandemic phase		
		2017.01.01 to 2019.12.31	2020.01.01 to 2022.12.31		
Out-of-hospital cardiac arrest	n = 925	n = 458	n = 467		
Insertion of a laryngeal tube	459 (50%)	155 (34%)	304 (65%)	< 0.0001	χ^2^=89.103
Establishment of secure venous access	493 (53%)	257 (56%)	236 (51%)	0.09	χ^2^=2.6708
Intravenous administration of adrenaline	279 (30%)	120 (26%)	159 (34%)	0.01	χ^2^=6.3905
Duration from initial patient contact to laryngeal tube insertion, min	3 (0-32)	0 (0-28)	4 (0-32)	< 0.0001	W=16587
Duration from initial patient contact to securing venous access, min	11 (1-37)	9 (1-37)	12 (2-37)	< 0.0001	W=19446
Duration from initial patient contact to adrenaline administration, min	14 (3-39)	12 (3-36)	16 (4-39)	< 0.0001	W=6536
Location of venous access establishment					
On-scene	123 (25%)	83 (32%)	40 (17%)	0.0001	χ^2^=14.665
Post-EMS arrival, pre-transport	192 (39%)	97 (38%)	95 (40%)	0.63	χ^2^=0.22918
Post-EMS arrival, during transport	178 (36%)	77 (30%)	101 (43%)	0.01	χ^2^=9.1346
Location of adrenaline administration					
On-scene	74 (27%)	45 (38%)	29 (18%)	0.0005	χ^2^=12.049
Post-EMS arrival, pre-transport	54 (19%)	21 (18%)	33 (21%)	0.54	χ^2^=0.27902
Post-EMS arrival, during transport	151 (54%)	54 (44%)	97 (61%)	0.01	χ^2^=6.4264
Prehospital return of spontaneous circulation	65 (7.0%)	31 (7%)	34 (7%)	0.86	χ^2^=0.030951
Outcomes					
CPC 1	6 (0.6%)	2 (0.5%)	4 (0.8%)		
CPC 2	4 (0.4%)	2 (0.5%)	2 (0.4%)		
CPC 3	9 (0.9%)	6 (1.3%)	3 (0.6%)		
CPC 4	8 (0.8%)	6 (1.3%)	2 (0.4%)		
CPC 5	888 (97.3%)	433 (96.7%)	455 (97.8%)		

Multivariate logistic regression analysis showed that bystander CPR (adjusted odds ratio (aOR): 1.68; 95% confidence interval (CI): 1.1-2.55) and prehospital time from ECI to OSA (aOR: 2.06; 95% CI: 1.36-3.11) significantly increased during the pandemic phase (Table 3). The likelihood of multiple hospital admission requests by EMS increased significantly during the pandemic phase (aOR: 9.5; 95% CI: 2.78-32.7). Laryngeal tube insertion (aOR: 3.2; 95% CI: 2.1-4.9) and initiation of venous access after transport also increased during the pandemic phase (aOR: 1.67; 95% CI: 1.06-2.63).

**Table 3:** Multivariate logistic regression analysis of out-of-hospital cardiac arrest event characteristics and outcomes between the coronavirus disease 2019 pandemic and pre-pandemic phases Abbreviations: OHCA, out-of-hospital cardiac arrest; CPR, cardiopulmonary resuscitation; ECI, emergency call intake; OSA, on-scene arrival; PL, patient loading into ambulance; DS, departure from the scene; EMS, emergency medical services

		Binary logistic regression analysis			Multivariate logistic regression analysis	
Variable	Events vs. Reference Level	Pandemic vs. pre-pandemic			Pandemic vs. pre-pandemic	
		Unadjusted Odds Ratio (95% confidence interval)	P-value		Adjusted Odds Ratio (95% confidence interval)	P-value
Pulseless electrical activity	Yes vs. No	0.84 (0.51-1.39)	0.49			
Bystander CPR performed	Yes vs. No	1.65 (1.1-2.5)	0.02		1.68 (1.1-2.55)	0.01
Witnessed arrest	Yes vs. No	1.00 (0.62-1.62)	0.98			
Prehospital time from ECI to OSA	≥ 10 min vs. < 10 min	2.05 (1.35-3.11)	< 0.001		2.06 (1.36-3.11)	< 0.001
Prehospital time from PL to DS	≥ 6 min vs. < 6 min	1.16 (0.69-1.92)	0.57			
Two or more inquiries about hospital admission requests via EMS	Yes vs. No	8.9 (2.5-31.3)	< 0.001		9.5 (2.78-32.7)	< 0.001
Insertion of a laryngeal tube	Yes vs. No	3.23 (2.09-4.99)	< 0.001		3.2 (2.1-4.9)	< 0.001
Establishing venous access during transportation to the hospital by EMS	Yes vs. No	1.79 (1.1-2.93)	0.01		1.67 (1.06-2.63)	0.02
Prehospital return of spontaneous circulation	Yes vs. No	1.02 (045-2.32)	0.95			

## Discussion

During the COVID-19 pandemic, delays in EMS response times, specifically from ECI to OSA and from ECI to ATH, have been reported globally, including in Japan [1,7-9]. Notably, this is the first study to analyze the total prehospital time in detail across the pandemic phase and evaluate shifts in the timing of EMS interventions. Previous reports from Japan indicated a significant decrease in favorable neurological outcomes in patients with OHCA after the onset of the pandemic [3]. However, we observed no increase in OHCA incidence during the study period. Moreover, there were no differences in factors such as bystander-witnessed arrest, initial rhythm, prehospital return of spontaneous circulation, or cerebral performance category.

In our study, bystander CPR rates increased, whereas previous reports have shown variations in bystander CPR rates under pandemic conditions, depending on the country or region [3,8,9,13-16]. A report indicating a decline in bystander CPR in Japan pertains mainly to densely populated urban areas [3]. However, reports often attribute increased bystander CPR to the increased presence of family members at home due to stay-at-home orders [9]. Our study area, a regional city with a high proportion of family-based households, likely experienced an increase in bystander CPR because of the presence of household members despite no increase in witnessed cardiac arrests. This increase may be attributed to the presence of family members at the time of the incident owing to home isolation practices.

The extended total prehospital time observed during the COVID-19 pandemic phase can partially be attributed to the increased time from ECI to OSA. Factors likely contributing to this delay include the additional precautions to prevent COVID-19 transmission, such as confirming potential infection or contact status before dispatch and donning personal protective equipment [1,2,16]. In our region, the increased number of ambulance dispatches also led to cases in which no nearby units were available, requiring dispatch from distant locations. The extension of the time from PL to DS was influenced by a complex interaction of factors, including increased hospital admission requests and changes in EMS intervention content and timing.

During the pandemic phase, adrenaline administration increased significantly. This was likely due to the prolonged activity time after patient contact, which provided opportunities for adrenaline administration before arrival at the hospital. A notable decrease in on-scene venous access and adrenaline administration and increased venous access during transport was observed. This change may be due to the additional time required for laryngeal tube insertion, the extended time needed to select an appropriate hospital, and the preference for performing procedures in the ambulance, which was perceived to be safe regarding COVID-19 exposure.

COVID-19 primarily spreads through droplets and direct contact. However, aerosol transmission should also be considered. Aerosol transmission risk is high in poorly ventilated indoor environments [17] and during procedures such as intubation [18]; however, effective ventilation and face mask use can reduce the infection risk by 5-10 times [17]. Personal protective equipment, including N95 masks, is highly effective, and with proper protocols, EMS personnel face minimal risk of COVID-19 from patient contact [19]. Thus, with appropriate infection control measures, EMS personnel may be protected from patient transmission. This suggests that resuscitation priorities focused on airway management to minimize aerosol exposure should be reconsidered in favor of early on-scene adrenaline administration.

In this study, the time from patient contact to adrenaline administration was 12 minutes and 16 minutes during the pre-pandemic and pandemic phases, respectively. Notably, numerous studies have shown the benefits of early adrenaline administration in patients with OHCA [20-24]. Therefore, given that adrenaline should be administered within 10 minutes of patient contact for optimal outcomes [23] and that every one-minute delay in initial adrenaline administration reduces survival by 4% [24], the observed four-minute delay in adrenaline administration during the pandemic phase, with increased reliance on venous access during transport, is a significant concern. Thus, prioritizing airway management with devices as the primary intervention after patient contact may require reassessment until 2024. Moreover, the current procedural structure requiring specific instructions may contribute to delayed adrenaline administration. Studies have indicated that streamlining instructions can save up to 30 seconds [25]. Therefore, implementing generalized instructions, as observed in other countries, could be a method to reduce delays.

Our study has some limitations. This study was conducted in a single regional city in Japan, with a population of approximately 170,000, and the findings may not be directly applicable to other regions. Additionally, data were primarily collected on EMS-related variables in the prehospital setting, with limited information on patient background, such as comorbidities or family structure, and hospital-based treatment details. Notably, differences in post-transport treatment and post-resuscitation care could have affected the outcomes; however, such data were unavailable in this study.

## Conclusions

In conclusion, during the COVID-19 pandemic, EMS response times to arrive at the scene were delayed, and the frequency of hospital admission requests increased, posing operational challenges. A shift in operational policies has increased reliance on venous access establishments during transport. Therefore, when managing OHCA amid emerging infectious disease outbreaks, developing response strategies that address delays in adrenaline administration is essential.
